# Strategies for Intraspecific Genotyping of Duckweed: Comparison of Five Orthogonal Methods Applied to the Giant Duckweed *Spirodela polyrhiza*

**DOI:** 10.3390/plants11223033

**Published:** 2022-11-09

**Authors:** Manuela Bog, Luca Braglia, Laura Morello, Karen I. Noboa Melo, Ingo Schubert, Oleg N. Shchepin, K. Sowjanya Sree, Shuqing Xu, Eric Lam, Klaus J. Appenroth

**Affiliations:** 1 Institute of Botany and Landscape Ecology, University of Greifswald, 17489 Greifswald, Germany; 2 Istituto Biologia e Biotecnologia Agraria, Via Bassini 15, 20131 Milano, Italy; 3 Leibniz Institute of Plant Genetics and Crop Plant Research (IPK), Gatersleben, 06466 Stadt Seeland, Germany; 4 Department of Environmental Science, Central University of Kerala, Periye 671320, India; 5 Institute of Organismic and Molecular Evolution, Johannes Gutenberg University Mainz, 55128 Mainz, Germany; 6 Department of Plant Biology, Rutgers the State University of New Jersey, New Brunswick, NJ 08901, USA; 7 Matthias Schleiden Institute—Plant Physiology, University of Jena, 07743 Jena, Germany

**Keywords:** *Spirodela polyrhiza*, Lemnaceae, duckweed, genotyping, intraspecific variation

## Abstract

The predominantly vegetative propagating duckweeds are of growing commercial interest. Since clonal accessions within a respective species can vary considerably with respect to their physiological as well as biochemical traits, it is critical to be able to track the clones of species of interest after their characterization. Here, we compared the efficacy of five different genotyping methods for *Spirodela polyrhiza*, a species with very low intraspecific sequence variations, including polymorphic NB-ARC-related loci, tubulin-gene-based polymorphism (TBP), simple sequence repeat variations (SSR), multiplexed ISSR genotyping by sequencing (MIG-seq), and low-coverage, reduced-representation genome sequencing (GBS). Four of the five approaches could distinguish 20 to 22 genotypes out of the 23 investigated clones, while TBP resolved just seven genotypes. The choice for a particular method for intraspecific genotyping can depend on the research question and the project budget, while the combination of orthogonal methods may increase the confidence and resolution for the results obtained.

## 1. Introduction

In the last two decades, the evolution of molecular methods has revolutionized phytotaxonomy. These advances have also been applied to the duckweed family, Lemnaceae Martinov [1,2]. The taxonomic investigation of duckweeds using molecular methods started with the genotyping work presented in [3] and was extended with the application of several methods, such as amplified fragment length polymorphism (AFLP) and plastidic and nuclear barcoding (reviewed in [4]). Classification based on morphological markers was the sole option of botanists for centuries (for duckweeds, see [5,6]) and was, to a great extent, confirmed and extended by molecular taxonomic investigations, uncovering phylogenetic relationships. It should be emphasized that there is little in common between these two approaches, with the latter being a more quantitative method.

Presently, 30 out of 36 species of duckweed can be reliably identified by molecular taxonomy [7,8]. Thus, further progress in these methods is still required. Several areas of duckweed research require not only the identification of species but also that of specific clones from the same species, since some physiological properties of duckweeds are defined at the level of clones rather than the level of species and are sometimes linked with the respective ploidy level [9]. Such features include, e.g., the growth rate [10,11] and starch accumulation under the condition of the deficiency of essential nutrients, such as nitrate, phosphate, and sulphate [12], and other stresses [13,14]. Furthermore, in view of the growing commercial perspective of duckweeds, the patenting of specific clones [15] or their monitoring in commercial products would also require the ability to distinguish clones of interest from others of the same species. However, identification at the level of clones for duckweeds is extremely difficult using solely morphological markers due to their simple structure and abbreviated anatomical features. Therefore, suitable molecular methods for intraspecific genotyping must be developed.

**Figure 1:**
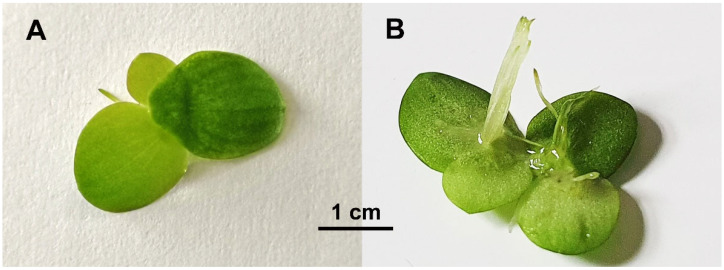
A colony of fronds of *Spirodela polyrhiza*, clone 7498: (**A**) dorsal view; (**B**) ventral view.

Among the duckweed family, *Spirodela polyrhiza* (L.) Schleid (commonly called greater duckweed, Figure 1) displays unusually low intraspecific genetic variation. This was first suggested by the authors of [16], who found that there is no significant difference in genome size between the 38 clones of this species (in contrast to several other duckweed species; see [17], this Special Issue), and the investigation of three plastidic regions similarly detected hardly any variations. Subsequently, clonal variations between two reference genome quality assemblies of *S. polyrhiza* clones were found to be very low in terms of both intraspecific sequence variations as well as heterozygosity [18], with the single-nucleotide polymorphism positions (SNPs) being approximately six times fewer than those found between the ecotypes of *Arabidopsis* [19,20]. Using a population genomics approach with low-coverage sequencing reads, refs. [21,22] further extended the generality of these characteristics by finding very low genetic diversity between *S. polyrhiza* clones from a large number of locations across the globe. Moreover, the genome-wide spontaneous mutation rate in this species was estimated to be seven times lower than those of other multicellular eukaryotes characterized to date [21]. Using a novel approach to systematically identify and rank the polymorphic loci in the nuclear genome that may enable effective intraspecific genotyping, the authors of [23] used the genome sequences (40X or more coverage) from 10 clones of greater duckweed as a training set to identify loci among the NB-ARC-related gene family that could be used to discriminate between the clones of this species. This gene family, which is known to be involved in plant defence and immunity, was chosen because it displays the highest intraspecific polymorphism among the plant genomes that have been studied [19,24]. Validated primer sets were then used to uncover the intraspecific variations with an additional 13 clones of *S. polyrhiza*, bringing the total number to 23. From this work, 20 genotypes of these 23 clones could be distinguished. All 23 clones were selected from the list of 36 that were studied in [25] for their specific turion yield trait, based on their availability in the RDSC at the time. Three of the tested clones could not be resolved from each other using this genotyping technique but were distinct from each other in terms of the specific turion yield [25]. Nevertheless, the application of NB-ARC-related gene polymorphism represented a leap forward for the identification of intraspecific variations in Lemnaceae.

Here, we used the same clones as those studied by [23] for comparative studies using four additional orthogonal methods: fragment length polymorphisms (FLPs) and/or single-nucleotide polymorphisms (SNPs), tubulin-gene-based polymorphism (TBP; [26]), simple sequence repeat variants (SSR; [27]), multiplexed ISSR genotyping by sequencing (MIG-seq; [28]), and genotyping by genome-wide sequencing (GBS; [7]). In all five cases, we calculated dissimilarity trees to evaluate the different methods in terms of their genotyping efficacy and considered the advantages and limitations of each.

## 2. Results

### 2.1. NB-ARC-Related Genes (NB-ARC)

**Figure 2:**
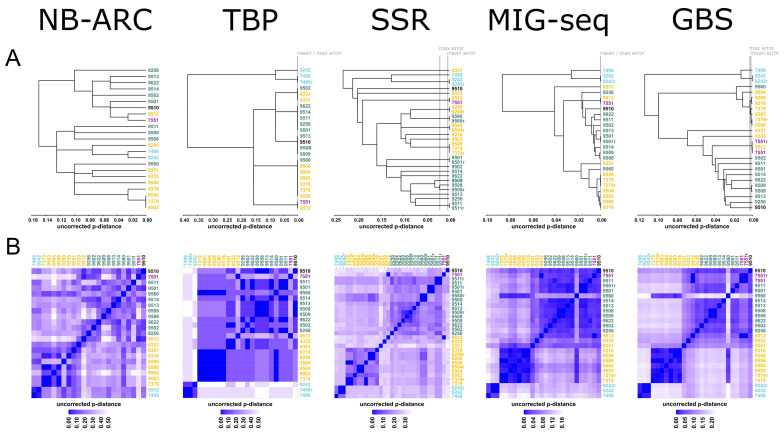
Comparison of five molecular methods for the genotyping of 23 clones of *S. polyrhiza*, including replicates (denoted by r). Colours denote the continent of origin of the clones: black—Africa, light blue—North + South America, yellow—Asia, violet—Australia, blue green—Europe. Upper panel (**A**) single-linkage clustering based on the uncorrected p-distances. The borders of resolution are given by the mean and max errors, calculated from the replicated clones. Lower panel (**B**) heat map representation of the uncorrected p-distances.

The NB-ARC approach is based on FLPs and SNPs of the DNA samples after their amplification with different primer sets, the sequences for which were generated and ranked by an informatic pipeline targeting genomic loci containing annotated NB-ARC genes [23]. Overall, 40 polymorphic characters were observed, with 17 based on FLPs and 23 based on SNPs (Table 1). The ambiguous, i.e., non-homozygous differences amounted to 26%. Figure 2 shows a single-linkage cluster and a heat map representation based on the uncorrected p-distance for the 23 *S. polyrhiza* clones used. All the numeric data, including the absolute and relative number of differences between clones, are given in the Supplementary Data S1. Three clones, 7379, 9503, and 9506, form a single data point and could not be distinguished from each other, but they are distinct from the other 20 clones. Thus, 21 genotypes were resolved from the 23 clones using this method. It should be noted that no error calculation is available for this approach, but based on the experience of one of the authors (E.L.), the repetition of the investigation of the same clone always produces identical results. One advantage of this method, as well as the TBP method, is that the number of fragments per sample tested is relatively small for the FLP and SNP analysis. Thus, the problem that usually affects other generic approaches in terms of the ambiguity in the band assignment becomes less of an issue. In any case, the results of this work revealed a geographic pattern associated with the dendrogram, i.e., clones from one continent were more likely to be similar to each other than clones from different continents (Figure 2).

### 2.2. Tubulin-Gene-Based Polymorphism (TBP)

The TBP method uses FLPs from two introns of the conserved, multigene β-tubulin family. Since only a relatively small number of genomic loci were queried using this method, the number of variants available for the differentiation of the clones was also lowest when using this method. A total of 13 characters (fragment lengths), including four monomorphic clones, were detectable, with all of them originating from the first intron and with no polymorphism scored for the second intron (Table 1, Supplementary Data S1). Since the fragments were scored as either present or absent, no ambiguous character was detected. Due to the low number of the polymorphic scores, the resolution of this method for the 23 clones studied was very low, and only seven distinct genotypes could be resolved (Figure 2, Supplementary Data S1). The one sample that was run in replicates in this work yielded an identical result. The same was observed by two of the authors (L.B. and L.M.) for up to four replicates per sample of other *S. polyrhiza* clones not investigated in this study. In spite of the relatively low resolution of this method, a geographic pattern became obvious as well (Figure 2).

### 2.3. Simple Sequence Repeats (SSR)

The SSR method, like the TBP method, uses FLPs for the differentiation of different genotypes. In total, 95 amplification fragments resolved by their mobility on gels were scored as present or absent, which led, again, to no ambiguous characters (Supplementary Data S1). The error rate for the SSR was the highest of all the tested methods, but at 0.26%, it was still considered low (Table 1). Of the 23 investigated clones, 21 different genotypes could be detected. It was not possible to discriminate clone 7379 from clone 9506 and clone 7551 from clone 9512. A geographical pattern was, again, observable (Figure 2).

### 2.4. Multiplexed ISSR Genotyping by Sequencing (MIG-Seq)

The MIG-seq method is an SNP-based method. The final dataset (alignment can be found as Supplementary Data S2), chosen from the 20 tested parameter combinations, consisted of 1292 characters, of which 29% or, on average, 380 ± 69 characters per sample were ambiguous due to their heterozygosity and gaps (Supplementary Data S3). Surprisingly, there were no differences in homozygous position between the three samples that were run in replicates, which displayed an error rate of 0% (Table 1). The method revealed 20 genotypes. The clones 7379 and 9506, 7551 and 9512, and 9290 and 9316 could not be distinguished from one another. The geographical pattern was characterised by shorter branch lengths within the geographical subgroups rather than between the groups, in addition to the division between the continents of origin (Figure 2).

### 2.5. Genotyping-by-Sequencing (GBS)

The SNP-based GBS method yielded 6170 SNPs in total, which is the highest number of characters among all the tested methods (for the alignment, see Supplementary Data S4). The error rate (0.17%) and proportion of ambiguous characters (14%) are intermediate compared to the other methods (Table 1). Depending on the strictness of the error rate treatment, GBS could distinguish 22 genotypes (mean error rate as the threshold) or 17 genotypes (maximum error rate as the threshold) (Supplementary Data S1). Using the mean error rate as a threshold, only the clones 9506 and 9316 could not be distinguished from one another, while using the maximum error rate, the following clone pairs could not be distinguished from each other: 7551/9512, 7379/9506, 9290/9316, 9503/9506, 9506/9316, and 9509/9508. The clustering methods show a clear separation of the American clones from all the other clones (Figure 2). Additionally, the clones from Europe can be found in a separate cluster, although clone 9560 from Hungary clusters with the Asian clones, and this unusual pattern was found using all the investigated methods, including that of TBP. The Asian clones show a paraphyletic clustering, with clones 9333 (Hubei-China), 9351 (Hanoi-Vietnam), and 9512 (Irkutsk-Russia) being more similar to the clones from Europe and the one from Australia. The other monophyletic Asian clones are from India.

## 3. Discussion

In order to test the efficacy of the orthogonal molecular methods in distinguishing between the clones of *S. polyrhiza*, we compared the NB-ARC, TBP, SSR, MIG-seq, and GBS approaches. At least four of these methods are known to have a high capacity to distinguish genotypes. We added TBP in this work, because this method is experimentally easy to carry out, as just two PCR reactions, followed by capillary electrophoresis, are required. Moreover, the capacity of TBP for resolving certain clones of the same species has been successfully tested on different *Lemna* species [29].

In most cases, the tested methods detected 20 or 21 genotypes among the 23 investigated clones. By GBS, with a lower stringency error rate treatment, even 22 genotypes may be distinguishable. We were unable to discriminate between all 23 clones using any of these methods alone. Four methods failed to distinguish clones 7379 and 9506 from each other, while with GBS, at a higher stringency, these two clones also became difficult to resolve. The situation was almost the same for the pair of clones 9506 and 9503, where SSR was the only method able to resolve these two genotypes. All three of these clones with unusually high genome sequence identities, 7379, 9506, and 9503, are from India, but they exhibited a more than 3-fold range difference in their specific turion yields of 1.86, 0.97, and 0.51, respectively. We therefore suspect that relatively specific genomic sequence (or epigenome) variations might account for these trait differences, consistent with previous speculations [23]. Our data were analysed quite strictly, and only the homozygous sites were considered for the method comparison. This led to a weakening of the results, because even heterozygous differences could represent genotype-distinguishing features. Nevertheless, we decided to opt for this strict approach, which does not require any complicating assumptions about phasing.

As expected, the methods that yield a high number of total characters and, therefore, differences, i.e., MIG-seq and GBS, showed the best resolution, especially in the phylogeographical studies. The geographical pattern closely fitted the results obtained by whole-genome resequencing in [21], which also separated the Indian *S. polyrhiza* clones from the Southeast Asian ones and clustered clone 9560 from Hungary together with the Asian clones. A great advantage of these two methods is their easy applicability for a wide range of organisms, although both methods require Illumina sequencing and the related equipment [7] (see Table 2 for a general comparison of the applied methods). The costs, especially if a company is asked to perform the analyses, are relatively high compared to the other methods. Furthermore, MIG-seq has an advantage over GBS, because in the case of MIG-seq, degraded DNA can be used as a template, since the amplified fragments between the SSR-based primers are relatively short. In contrast, GBS requires very good-quality DNA, since the first step is a fragmentation using restriction enzymes, and the clustering success of the single short Illumina-sequenced fragments highly depends on the quality of the template.

The NB-ARC polymorphism-based genotyping approach has shown a very good performance in genotyping *S. polyrhiza* [23], as observed here, with many of the original key observations confirmed and extended by the present work. A drawback of this method is the genome assembly resource required at the outset to establish this marker system for the species of interest. A well-assembled reference genome for the species and the nine other sequenced accessions were used as a training set for *S. polyrhiza*, a species known to have unusually low sequence variations, in order to informatically generate a ranked list of suitable primers to amplify the most polymorphic regions among the NB-ARC genes in the genome [23]. To improve the resolving power of this method, one could include additional genome sequence data on challenging clones, such as 7379 and 9503, in our training set for the pipeline, since clone 9506 was already part of the original training set. One apparent limitation of this approach could affect genomes that have a limited number of NB-ARC genes, such as the recently sequenced *Wolffia australiana* genome that has only three to four copies of these genes remaining [30]. In these cases, other large-plant gene families with members containing highly conserved exons could be used in the same informatic pipeline as that established in [23] to generate the requisite primers. For example, we note that in *W. australiana*, there is an amplification of the genes encoding the LRR-RK type of pattern recognition receptors (PRRs) to about 90 copies [1]. This gene family may present an alternative to the NB-ARC genes in *Wolffia australiana* for the generation of intraspecific genotyping primer sets.

SSR is likewise powerful, but it requires at least partial genomic information as well as the selection of useful sequence repeats and the design of primers in the flanking regions, as conducted in [27]. Furthermore, the use of cross-amplification to find useful polymorphic sites in related species is rather inefficient. The authors of [27] demonstrated this for *Lemna perpusilla*, and tests on a clone of *S. intermedia* (the sister species of *S. polyrhiza*) were unsuccessful in the lab of one of the authors (M.B.). The screening of already available whole-genome sequences or shotgun genome information appears to be a straightforward method for developing SSR markers using tools such as GMATA and others ([31] and references therein). Once the proper primers are available, the experimental procedure is not highly demanding financially or with respect to the required equipment or the quality of the template DNA.

The TBP method yielded, in our analysis, only seven genotypes based on the 13 scored characters for the 23 *S. polyrhiza* clones studied here. The same method, however, provided a useful resolution for the presumed *Lemna minor* clones, with 34 characters scored for the first intron and 36 genotypes identified among the 40 analysed clones [32]. Successful intraspecific genotyping was previously reported in crop species such as grape and olive [33,34]. However, because *S. polyrhiza* generally shows very little intraspecific variation [21,22], this species is a tough match for the TBP method and could be an exception rather than the common rule. Thus, for a duckweed species that displays greater intraspecific genome variations, this simple approach could still be a useful genotyping tool.

In summary, we presented five methods, four of which are well-suited for genotyping *S. polyrhiza* and likely other species, even those beyond the duckweed family, with a low intraspecific variability. The decision regarding which of these methods should be applied depends on the question to be solved (and on the available budget and genome resources). In addition, while the data generated using the four most effective approaches are consistent, to a large extent, and thus provide strong support for their validity, they also suggest the potential advantage of combining two of these orthogonal methods to extend the power of the analysis. For example, using GBS analysis, under a lower stringency of error, only one pair of clones, 9506 and 9316, could not be resolved. These two clones, however, can be distinguished using any of the other three methods. Thus, if data based on GBS and one of these methods can be integrated together in a genotype matrix, the resolution of all 23 clones tested here could become feasible.

## 4. Materials and Methods

### 4.1. Plant Material and Cultivation

All 23 clones of *Spirodela polyrhiza* were acquired from the duckweed collection of the Matthias Schleiden Institute—Plant Physiology, University of Jena, Germany, and are available as living materials from the duckweed collections in Jena and at Rutgers State University of New Jersey (New Brunswick, NJ, U.S.A.) under the international four-digit code [35] given in Table 3. They were selected by the authors of [23] according to their geographical diversity and the wide range of their specific turion yield [25]. The clones were additionally characterised previously by measurements of their relative growth rate [11] and genome size [16], or these parameters were measured in the present project (Table 3). The species’ identity was confirmed by barcoding using the plastidic markers *rpl16*, *rps16*, and *atpF-atpH* [16]. In total, 20 out of the 23 clones were whole-genome sequenced [21,23,36]. All the plants were cultivated under axenic conditions in N medium under standardised conditions for 10 days [37]: 8 mM KNO_3_, 0.15 mM KH_2_PO_4_, 1 mM MgSO_4_, 1 mM Ca(NO_3_)_2_, 5 μM H_3_BO_3_, 0.4 μM Na_2_MoO_4_, 13 μM MnCl_2_, and 25 μM Fe(III)NaEDTA. The plant material was stored at −80 °C for further use.

### 4.2. DNA Isolation and Downstream Lab Work

DNA was isolated from 100–200 mg fresh weight of duckweed plants using the CTAB method [38] and quantified using a NanoVue spectrophotometer (GE Healthcare Europe GmbH, Freiburg, Germany). For GBS, the DNA was extracted using silica columns, according to [39].

The wet lab methods used for the analysis by NB-ARC are described in [23].

TBP amplification was performed by targeting both the 1st and 2nd introns of the multigene β-tubulin family, using two universal primer pairs, Fex-Rex and Fin-Rin, respectively, and PCR conditions described in [29]. FAM-labelled amplified fragments were separated by capillary electrophoresis (CE) using a AB3500 Genetic Analyzer (Thermo Fisher Scientific Inc., Waltham, MA, USA). The numerical output of the CE was exported to Excel format, and the fragments were aligned by size across the samples, considering a one-nucleotide (± 0.5) approximation. The amplicon size was considered as a marker, and its presence/absence was scored in a binary matrix (1/0, respectively), which was used for the subsequent elaboration of the data. Both the TBP 1st and 2nd introns were scored. Further technical details about the data acquisition and analysis are reported in [26]. The sample 7498 was run in duplicate using the same DNA extract for the error estimation.

For the SSR investigations, a total of 12 SSR markers multiplexed in three sets of four primer pairs each were used, according to [27]. The forward primers were labelled with a fluorescence dye (set1: SP12[FAM], SP14[HEX], SP43[CY3], SP51[ROX]; set2: SP6[HEX], SP25[ROX], SP29[FAM], SP42[CY3]; set3: SP36[HEX], SP45[ROX], SP47[CY3], SP53[FAM]). PCR amplification was performed using a thermal cycler (SensoQuest GmbH, Göttingen, Germany) in a total volume of 10 μL containing 45 ng DNA template, 2.5 μL of 2 X KAPA 2G (Kapa Biosystems Pty Ltd., South Africa), and the final concentration of each primer (SP6[0.1 µM], SP12[0.2 µM], SP14[0.2 µM], SP25[0.2 µM], SP29[0.2 µM], SP36[0.1 µM], SP42[0.4 µM], SP43[0.2 µM], SP45[0.4 µM], SP47[0.4 µM], SP51[0.2 µM], SP53[0.1 µM]). The samples were amplified under the following conditions: 3 min at 95 °C, 35 cycles of 15 s at 95 °C, 35 s at 54 °C, and 30 s at 72 °C, and 5 min of the final extension at 72 °C. Subsequently, 2.5 µL of the diluted PCR products (set 1: undiluted, set 2: 1:10, set 3: 1:25) were mixed with 0.16 µL of GeneScan 500 LIZ size standard and 10.5 µL HiDi formamide (both Thermo Fisher Scientific). Finally, the fragment analysis was carried out using a 3130xl Genetic Analyzer (Thermo Fisher Scientific). Eight samples (7379, 9242, 9290, 9501, 9504, 9508, 9511, and 9560) from the same DNA extracts were run in duplicate for the error estimation.

For MIG-seq, 25 µL of each DNA extract at a concentration of 30 ng/µL was sent to LGC Genomics GmbH (Berlin, Germany) for analysis. Sequencing by synthesis was performed with a 150 bp paired-end read chemistry using an Illumina NextSeq500/550 device (Illumina Inc., San Diego, CA, USA). Three samples (7379, 9242, 9501) from the same DNA extracts were run in duplicate for the error estimation. The sequence reads were uploaded to the sequence read archive (SRA) [40], PRJNA888369, accession numbers: SRR21845253-SRR21845278.

For the GBS method, DNA was used for the construction of barcoded libraries, essentially performed as described in [41]. Sequencing by synthesis (single read, 1 × 10^7^ cycles, index read 8 cycles) using the Illumina HiSeq 2500 device was performed according to the protocols provided by the manufacturer (Illumina Inc.). Three samples (7379, 7551, and 9242) from the same DNA extracts were run in duplicate for the error estimation. The sequence reads were uploaded to the sequence read archive (SRA) [40], PRJNA888369, accession numbers: SRR21845363-SRR21845387 and SRR21845392.

### 4.3. Data Analysis

The dataset for the analysis by NB-ARC was taken from [23], and the scoring of the TBP fragments was performed as described in [26]. The fragment lengths based on the SSR analysis were scored using GeneMapper v5 (Thermo Fisher Scientific) and subsequently rounded using Tandem v1.09 [42]. Finally, the fragment length data were converted into a 0/1 matrix. For MIG-seq, demultiplexing and trimming of sequencing adapters and primers from the raw sequence reads were done by the sequencing facility. Around 8 million pre-processed paired-end reads (mean: 307.5 thousand reads per sample, SD: 45.4 thousand reads) were de novo assembled and analysed using the ipyrad v0.9.84 pipeline [43] run on the HPC Brain Cluster of the University of Greifswald (Supplementary Data S5). We optimized the core assembly parameters ‘clustering threshold for de novo assembly’ (ct) and ‘minimum depth for statistical base calling’ (md) by running the analysis with 10 different values of ct (0.81–0.99, with a step of 0.2) and two values of md (6 and 10). Following the method of [44], the datasets obtained with the parameters ct = 0.91 and md = 10 were determined as optimal for the downstream analyses (Supplementary Data S5). The SNP identification of the approximately 77 mio pre-processed single-end reads (mean: 3 mio reads per sample, SD: 600 thousand reads) for the GBS data was performed as described in [7], except for the fact that the *S. polyrhiza* genome available in [45] was used for the SNP mapping.

We attempted to make the data analysis for the comparison as similar as possible, although the different methods produced different data types, including FLP (scored as 0/1) and SNP data. Therefore, 0/1 data was recoded as c/a, and each of the five datasets was saved as a FASTA file, which was further processed using R v4.1.0 [46], with the additional libraries ape v5.6-2 [47], stringdist v0.9.8 [48], phangorn v2.10.0 [49], reshape2 v1.4.4 [50], and ggplot2 v3.3.6 [51]. In a first step, the uncorrected p distance and character differences were calculated using the ‘dist.hamming’ function. Ambiguous characters were not considered. Then, a single-linkage clustering was applied using the ‘hclust’ function. The resulting trees were saved in newick format and formatted in FigTree v1.4.4 [52]. Heat maps were plotted using the ‘ggplot’ function after the conversion of the distance matrices into a linear form using the ‘melt’ function. Ambiguous sites were counted using a custom python script.

## Figures and Tables

**Table 1:** Comparison of the five tested methods for the genotyping of *Spirodela polyrhiza*, concerning the number of characters obtained and the proportion of ambiguous (non-homozygous) characters.

	NB-ARC	TBP	SSR	MIG-Seq	GBS
Marker Type ^1^	FLP + SNP	FLP	FLP	SNP	SNP
Number of Characters	40 (17 + 23)	13	95	1292	6170
Percentage of Ambiguous Characters	26	0	0	29	14
Average Number of Ambiguous Characters per Sample ± Standard Deviation	11 ± 5	0 ± 0	0 ± 0	380 ± 69	894 ± 286
Error Rate % (Number of Replicates) ^2^	n.d. (0)	0.00 (1)	0.26 (8)	0.00 (3)	0.17 (3)

^1^ FLP—fragment length polymorphism; SNP—single-nucleotide polymorphism; ^2^ n.d.—not determined.

**Table 2:** Pros and cons of the five different methods used for genotyping of 23 *Spirodela polyrhiza* clones with a worldwide distribution.

	NB-ARC	TBP	SSR	MIG-Seq	GBS
DNA requirements	degraded works as well	degraded works as well	degraded works as well	degraded works as well	high quality necessary
Establishment	elaborate, genome sequence information needed	easy	elaborate, sequence information needed	easy	easy
Universality *	no, primers can be species-specific	yes	(no), low cross-amplification	yes	yes
Resolution capacity	high	low	high	high	high
Costs per sample	low to moderate, depends on whether genome information is available	low	low	moderate	high

* indicates whether the established method can be easily transferred between species. The sequence-guided approach and pipeline created for NB-ARC is indeed universal, but specific primers need to be established for each species. The same applies for SSR.

**Table 3:** Origin of investigated *Spirodela polyrhiza* clones and their specific properties.

Clone ID	Country	Area/State	Specific Turion Yield ^1^	Specific Growth Rate (h^−1^) ^2^	Genome Size (Mbp/1C) ^3^	Genome Sequencing ^4^
7379	India	Tamil Nadu	1.86 ± 0.26	0.315 ± 0.008	158 ± 3	n.d.
7498	USA	North Carolina	2.37 ± 0.27	0.401 ± 0.016	157 ± 2	+
7551	Australia	Northern Territory	n.d.	0.376 ± 0.009	n.d.	+
9242	Ecuador	Guayas	1.37 ± 0.12	0.456 ± 0.003	n.d.	+
9256	Finland	Uusimaa	3.73 ± 0.25	0.296 ± 0.011	160 ± 6	+
9290	India	Delhi	1.40 ± 0.13	0.299 ± 0.010	n.d.	+
9316	India	Rajasthan	1.22 ± 0.13	0.281 ± 0.009	159 ± 2	+
9333	China	Hubei	n.d.	0.311 ± 0.033	n.d.	+
9351	Vietnam	Hanoi	n.d.	0.367 ± 0.024	n.d.	n.d.
9501	Albania	Fieri	5.93 ± 0.03	0.357 ± 0.005	164 ± 4	+
9502	Ireland	Leinster	1.64 ± 0.14	0.337 ± 0.008	164 ± 5	+
9503	India	Rajasthan	0.51 ± 0.03	0.312 ± 0.007	170 ± 5	+
9504	India	Rajasthan	0.34 ± 0.03	0.284 ± 0.012	168 ± 6	+
9506	India	Telangana	0.97 ± 0.07	0.313 ± 0.005	161 ± 7	+
9508	Poland	Cracow	0.66 ± 0.04	0.360 ± 0.007	160 ± 4	+
9509	Germany	Thuringia	0.51 ± 0.07	0.323 ± 0.006	157 ± 2	+
9510	Mozambique	Maputo	0.98 ± 0.10	0.289 ± 0.008	n.d.	+
9511	Russia	Moscow	2.25 ± 0.11	0.381 ± 0.009	161 ± 5	+
9512	Russia	Irkutsk	2.92 ± 0.49	0.386 ± 0.005	156 ± 3	+
9513	Czech	Jindřichův Hradec	1.16 ± 0.04	0.324 ± 0.006	161 ± 5	+
9514	Austria	Viena	1.17 ± 0.16	0.333 ± 0.012	159 ± 5	+
9560	Hungary	Bekes	n.d.	0.367 ± 0.008	160 ± 6	+
9622	Germany	Baden-Württemberg	n.d.	0.310 ± 0.011	159 ± 5	+

^1^ [25], ^2^ [11] or measured in the present project, ^3^ [16], ^4^ [21,23,36], n.d.—not determined.

## Data Availability

All the Illumina sequence data based on MIG-seq and GBS are available from the Sequence Read Archive (https://www.ncbi.nlm.nih.gov/, accessed on 12 October 2022). The respective project numbers and accession numbers can be found in the main text. The raw data and distance matrices for the other methods are available as Supplementary Data within this article.

## Supplementary Materials

The following are available online at https://www.mdpi.com/article/10.3390/plants11223033/s1, Supplementary Data S1.xlsx (primer/adapter sequences and input data of the five investigated methods and calculated p-distance matrices with absolute number of differences and proportion of differences); Supplementary Data S2.fas (aligned SNP data from the MIG-seq analysis); Supplementary Data S3.xlsx (general statistics on MIG-seq results); Supplementary Data S4.fas (aligned SNP data from the GBS analysis); Supplementary Data S5.pdf (MIG-seq analysis script and results of ipyrad parameter optimisation).
