# Supplementary material for: Strategies for Intraspecific Genotyping of Duckweed: Comparison of Five Orthogonal Methods Applied to the Giant Duckweed Spirodela polyrhiza
Source: Plants (Basel). 2022 Nov 9;11(22):3033. doi: 10.3390/plants11223033 (PMC9696241; doi:10.3390/plants11223033)
Supplement: Supplementary file 1 [file plants-11-03033-s001.zip › Supplementary Data/Supplementary Data S5.pdf]

Parameter evaluation plots

Manuela Bog, Oleg Shchepin

06.09.2022

Fraction of loci inferred as paralogs and discarded by ipyrad across different clustering thresholds and minimum read depths

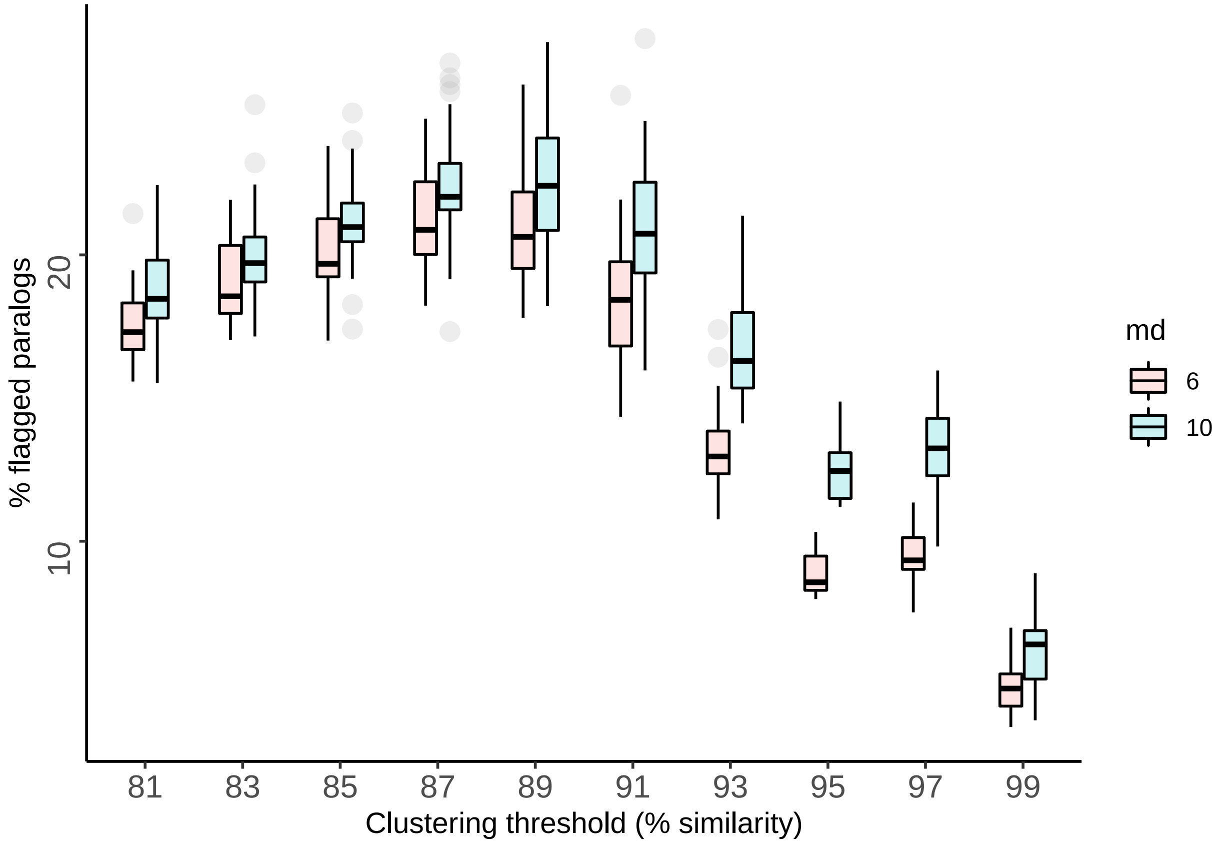

Heterozygosity across samples recovered across different clustering thresholds and minimum read depths

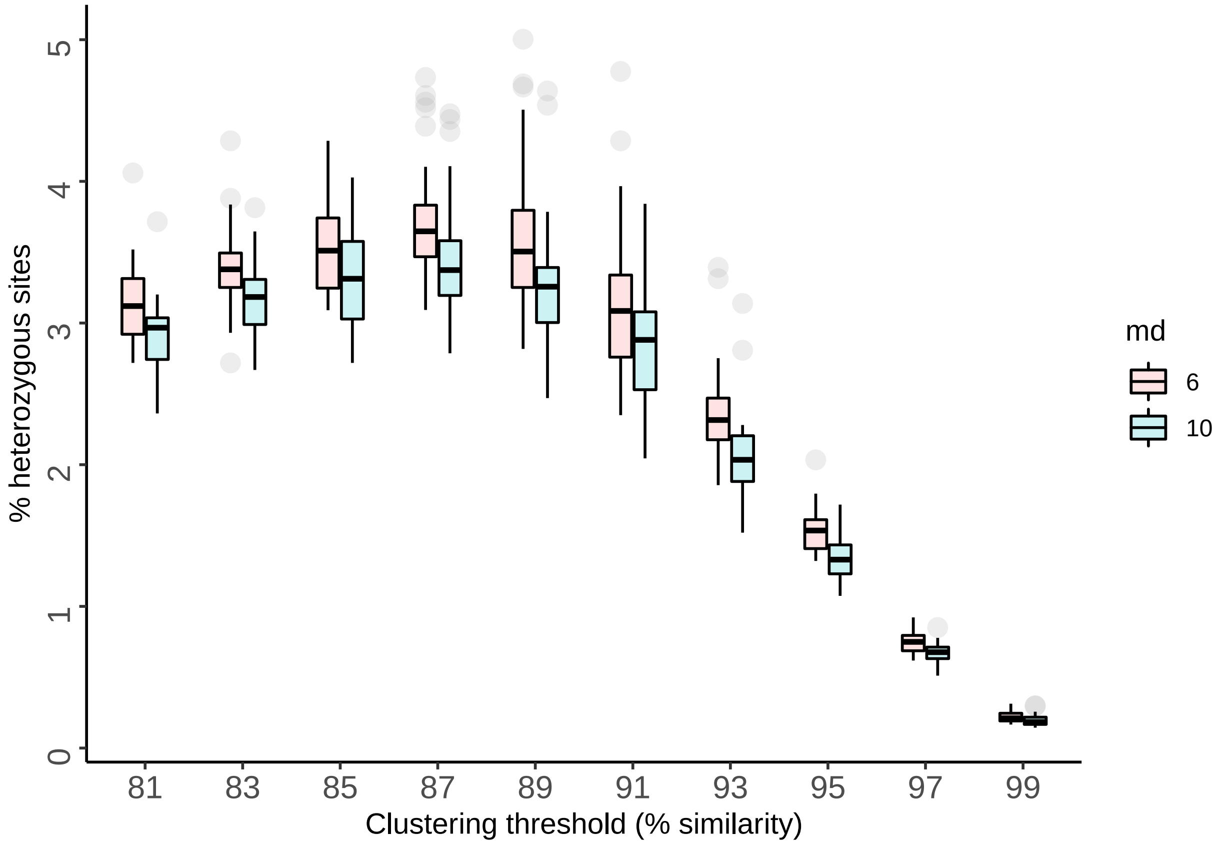

Total SNPs recovered across different clustering thresholds and minimum read depths

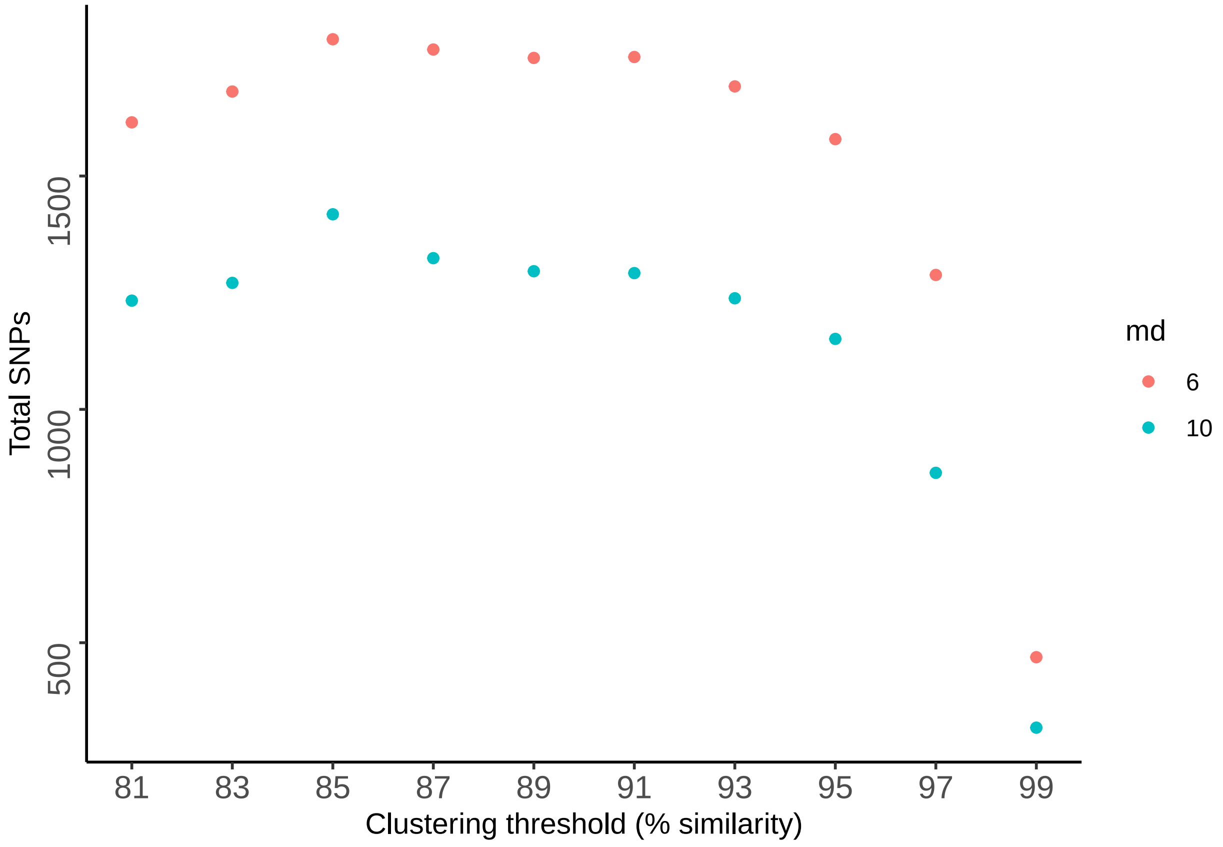

Cumulative variance of all biallelic SNPs recovered by ipyrad explained by the first five principal components across different clustering thresholds and minimum read depths

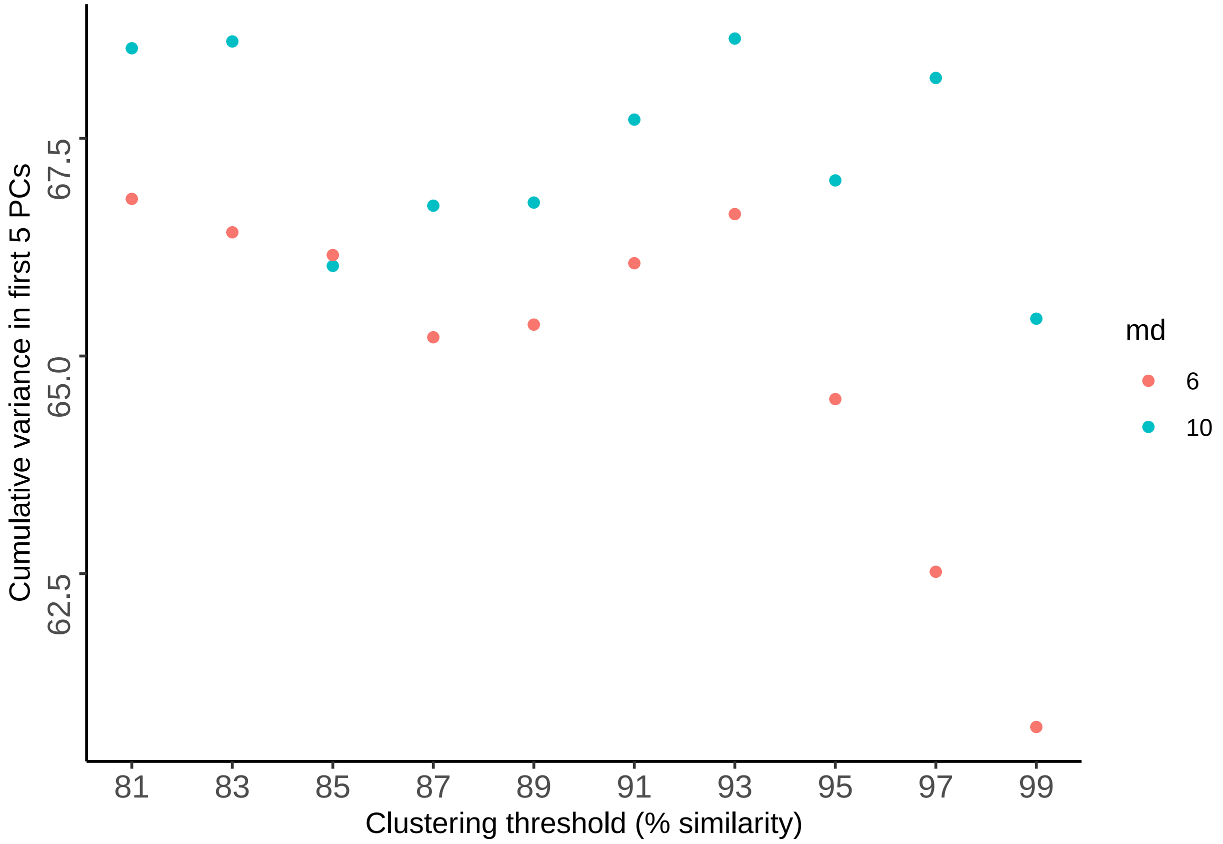

Pearson's correlation coefficient between pairwise genetic dissimilarity and data missingness at different clustering thresholds and minimum read depths

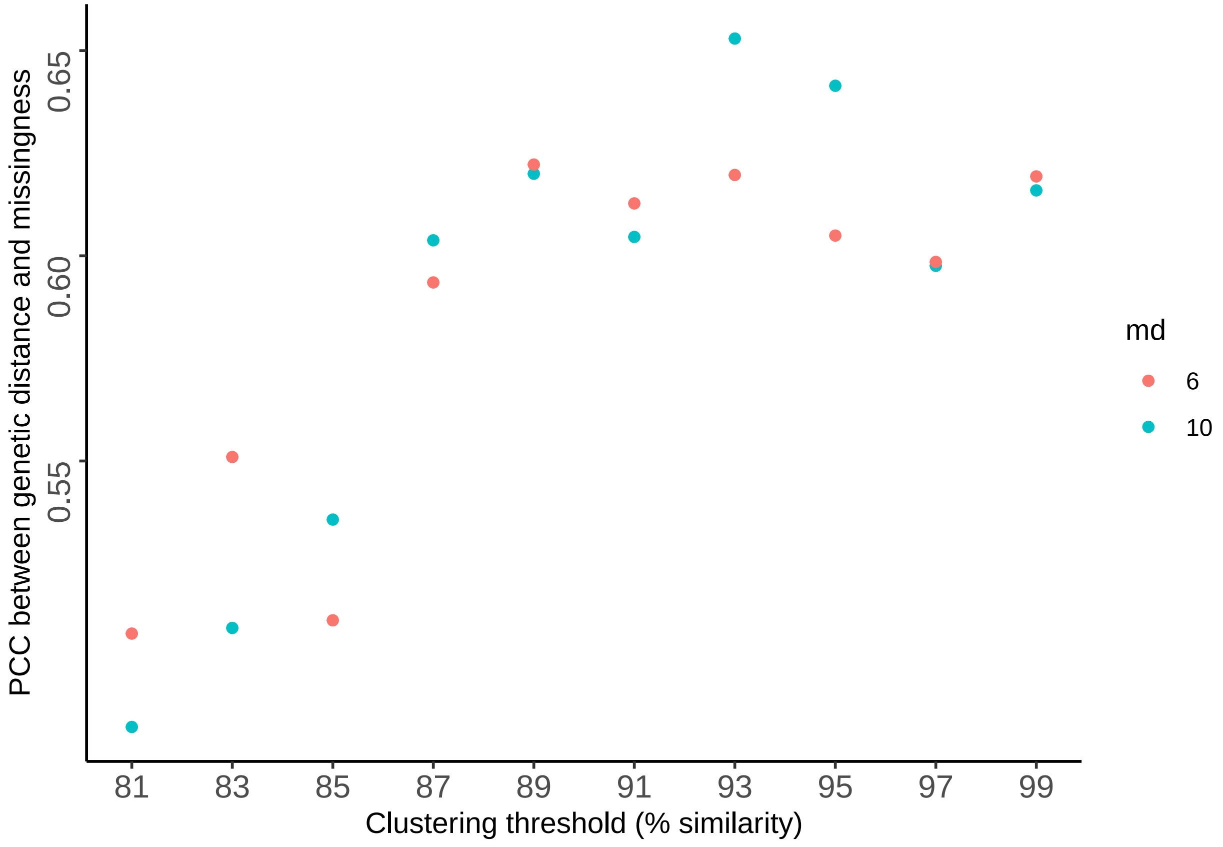

Total number of loci that contain at least one SNP recovered across different clustering thresholds and minimum read depths

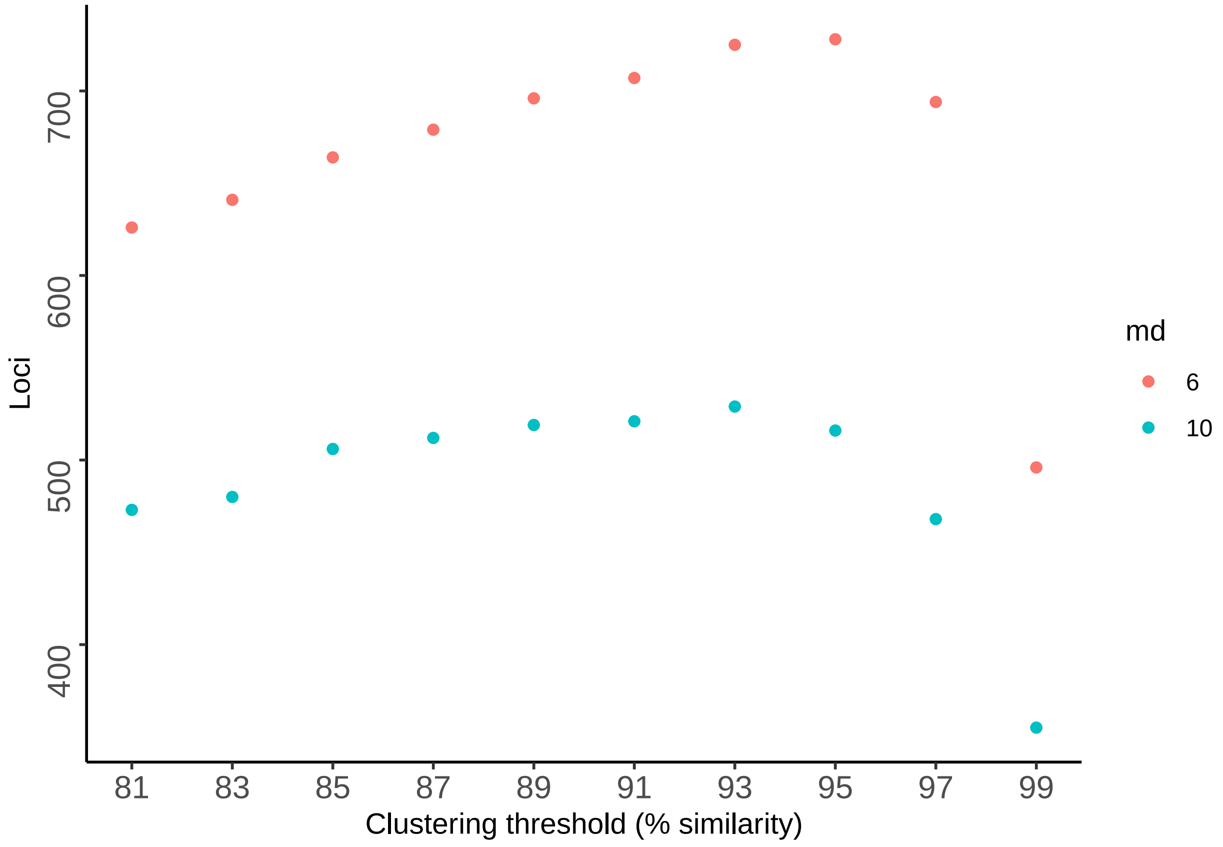

% of differences between replicated samples across different clustering thresholds and minimum read depths

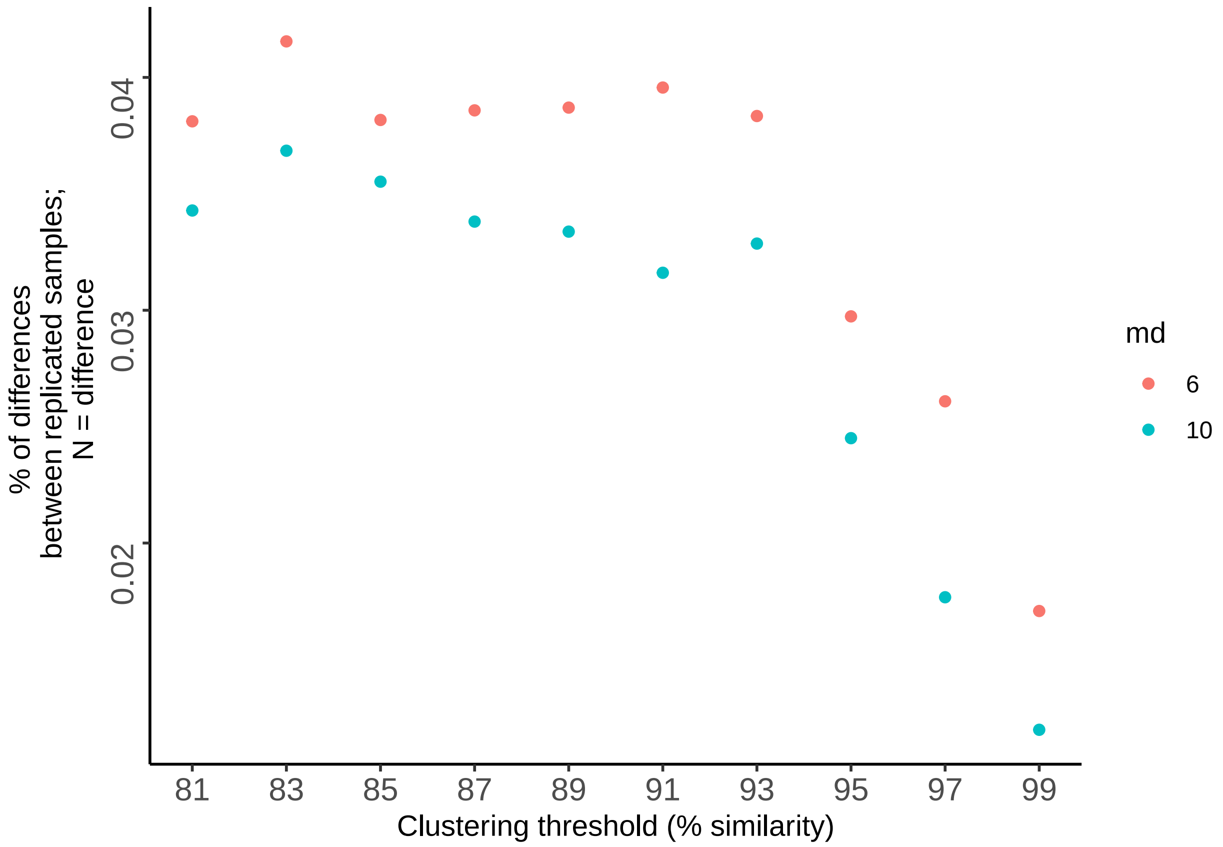

PCoA for clustering threshold 89% and minimum read depth 10

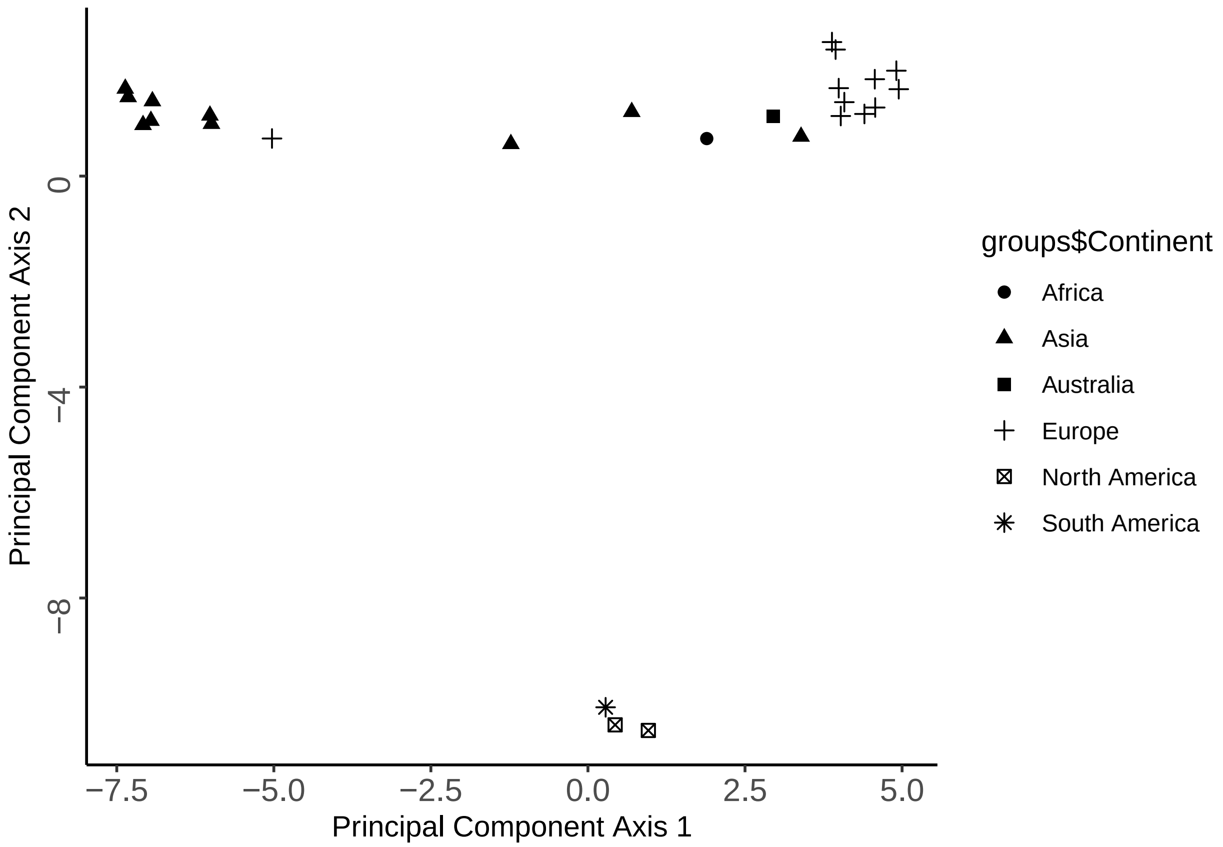

PCoA for clustering threshold 91% and minimum read depth 10

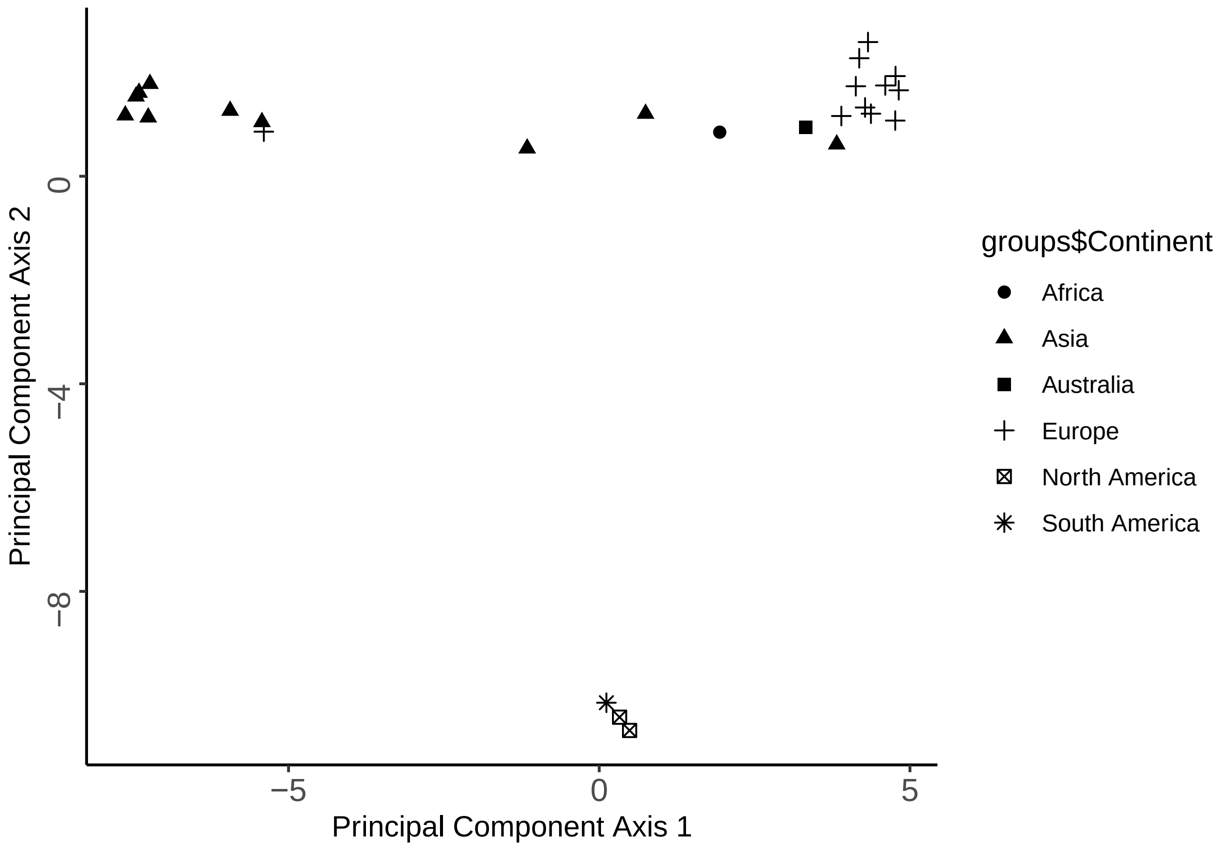

PCoA for clustering threshold 93% and minimum read depth 10

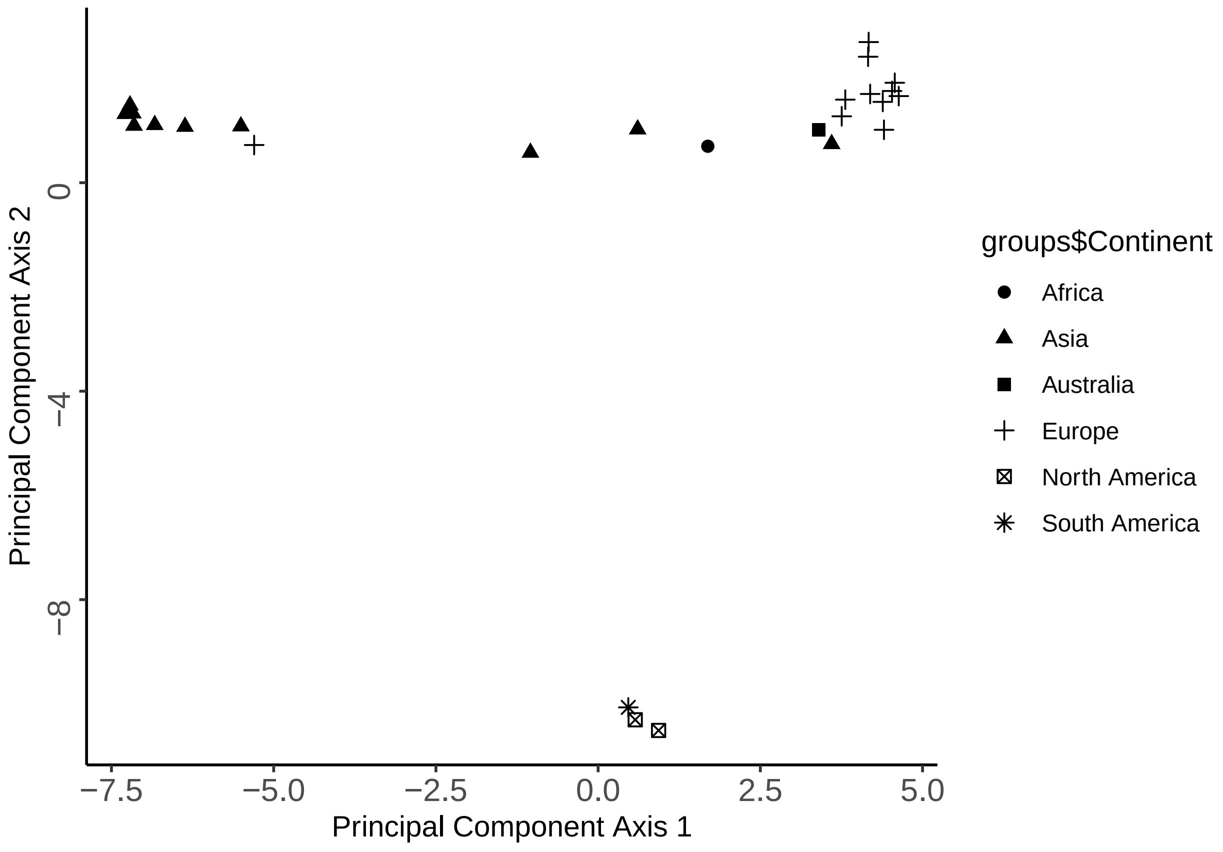

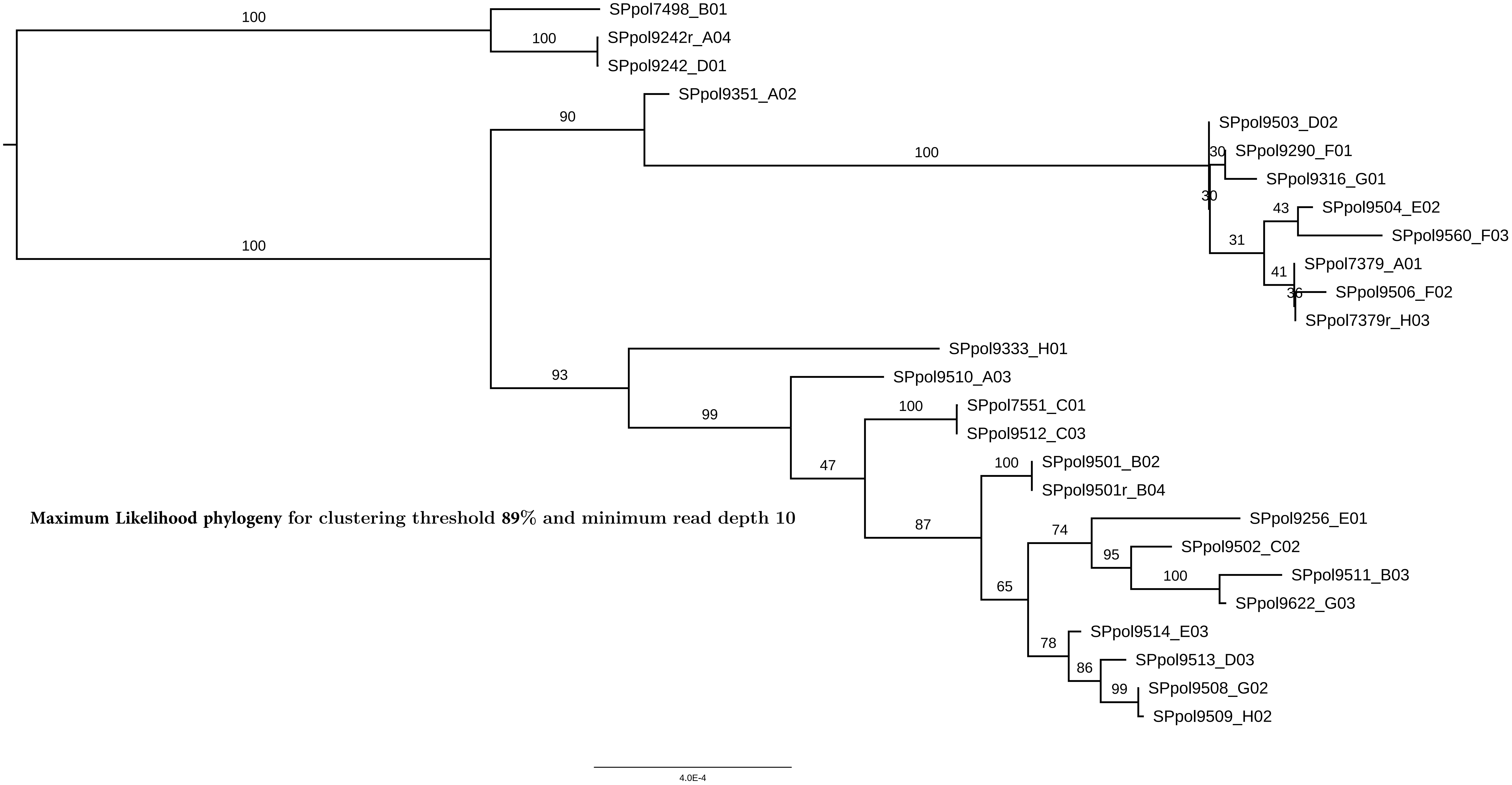

Maximum Likelihood phylogeny for clustering threshold 89% and minimum read depth 10

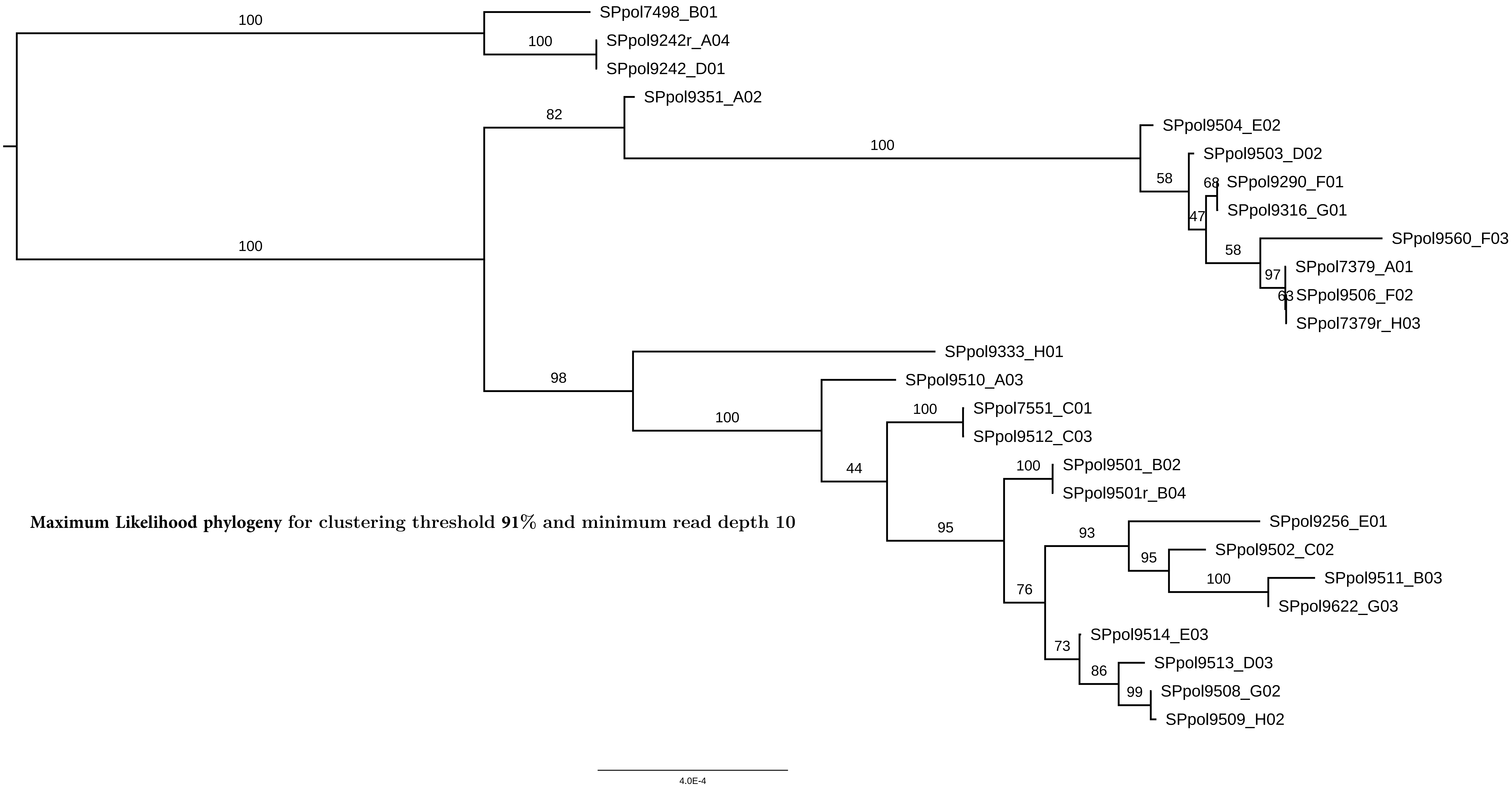

Maximum Likelihood phylogeny for clustering threshold **91%** and minimum read depth **10**

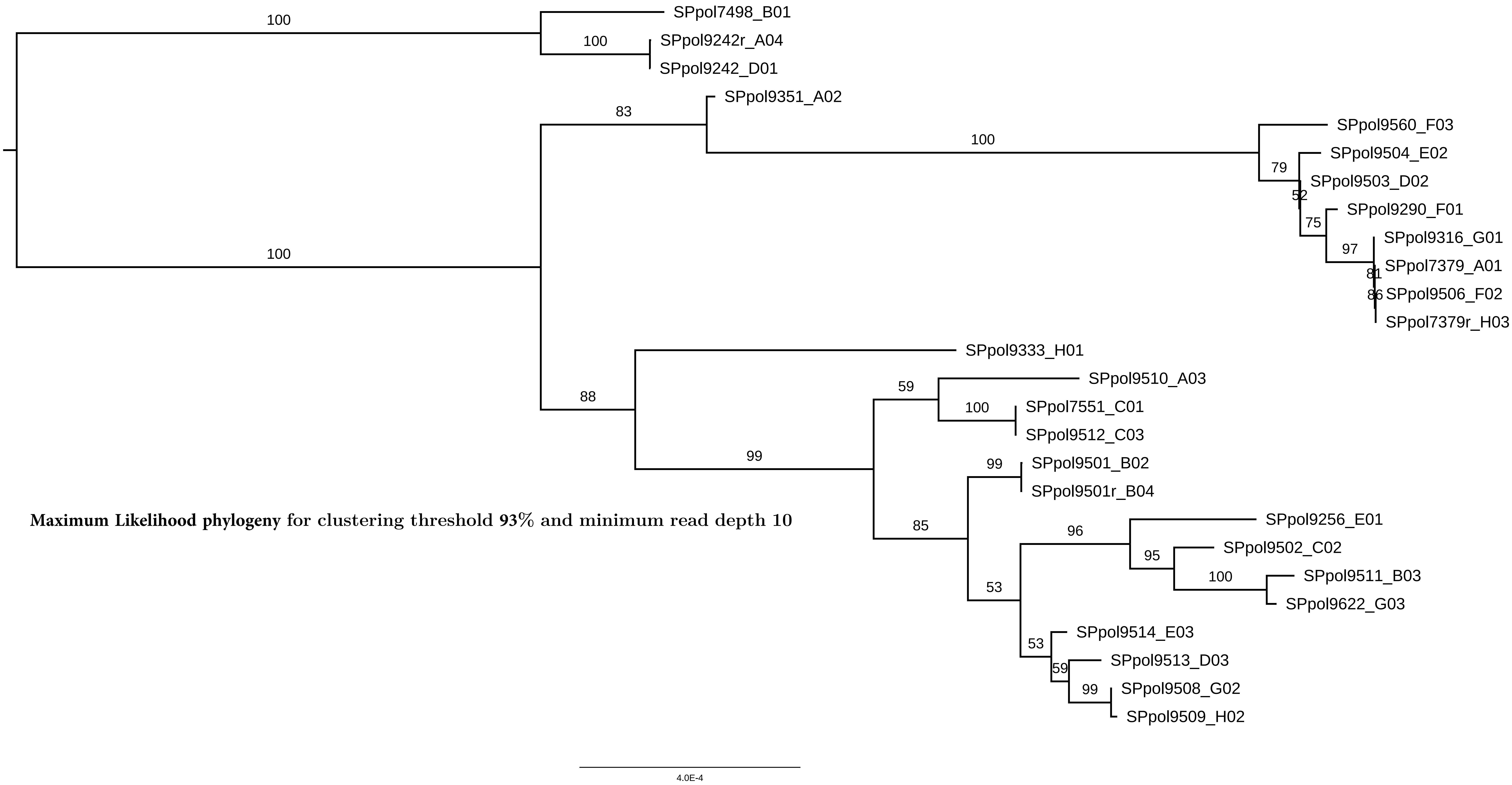

Maximum Likelihood phylogeny for clustering threshold 93% and minimum read depth 10

```
------- ipyrad params file (v.0.9.84)-------------------------------------------
md10ct91ms13                  ## [0] [assembly_name]: Assembly name. Used to name output directories for assembly steps
/home/shchepino/MIGseq/Spirodela_updated/output ## [1] [project_dir]: Project dir (made in curdir if not present)
                              ## [2] [raw_fastq_path]: Location of raw non-demultiplexed fastq files
                              ## [3] [barcodes_path]: Location of barcodes file
/home/shchepino/MIGseq/Spirodela/PrimerClipped/*.fastq ## [4] [sorted_fastq_path]: Location of demultiplexed/sorted fastq files
denovo                        ## [5] [assembly_method]: Assembly method (denovo, reference)
                              ## [6] [reference_sequence]: Location of reference sequence file
pairgbs                       ## [7] [datatype]: Datatype (see docs): rad, gbs, ddrad, etc.
TGCAG,                        ## [8] [restriction_overhang]: Restriction overhang (cut1,) or (cut1, cut2)
5                             ## [9] [max_low_qual_bases]: Max low quality base calls (Q<20) in a read
33                            ## [10] [phred_Qscore_offset]: phred Q score offset (33 is default and very standard)
10                            ## [11] [mindepth_statistical]: Min depth for statistical base calling
10                            ## [12] [mindepth_majrule]: Min depth for majority-rule base calling
10000                         ## [13] [maxdepth]: Max cluster depth within samples
0.91                          ## [14] [clust_threshold]: Clustering threshold for de novo assembly
0                             ## [15] [max_barcode_mismatch]: Max number of allowable mismatches in barcodes
2                             ## [16] [filter_adapters]: Filter for adapters/primers (1 or 2=stricter)
35                            ## [17] [filter_min_trim_len]: Min length of reads after adapter trim
2                             ## [18] [max_alleles_consens]: Max alleles per site in consensus sequences
0.05                          ## [19] [max_Ns_consens]: Max N's (uncalled bases) in consensus
0.05                          ## [20] [max_Hs_consens]: Max Hs (heterozygotes) in consensus
13                            ## [21] [min_samples_locus]: Min # samples per locus for output
0.2                           ## [22] [max_SNPs_locus]: Max # SNPs per locus
8                             ## [23] [max_Indels_locus]: Max # of indels per locus
0.5                           ## [24] [max_shared_Hs_locus]: Max # heterozygous sites per locus
0, 0, 0, 0                    ## [25] [trim_reads]: Trim raw read edges (R1>, <R1, R2>, <R2) (see docs)
0, 0, 0, 0                    ## [26] [trim_loci]: Trim locus edges (see docs) (R1>, <R1, R2>, <R2)
p, s, l, v, k, n              ## [27] [output_formats]: Output formats (see docs)
                              ## [28] [pop_assign_file]: Path to population assignment file
                              ## [29] [reference_as_filter]: Reads mapped to this reference are removed in step 3
```

Rmarkown script

```
---
title: "Parameter evaluation plots"
author: "Manuela Bog, Oleg Shchepin"
date: "06.09.2022"
output: pdf_document
---


```{r setup, include=FALSE}
knitr::opts_chunk$set(echo = FALSE)
Sys.setenv(LANG = "en")
```


```{r Libraries, include=FALSE}
# Load necessary libraries and select working directory


library(ggplot2)
library(Hmisc)
library(vcfR)
library(adegenet)
library(dartR)
library(ggplot2)
source("../utils.reset.flags.function.R", local = knitr::knit_global())

setwd("D:/Work/2022_Spirodela/ct_plots")

```



```{r Read_data, include=FALSE}


# Load and prepare the data for barplots and dotplots
```
```

```
cthet <- read.csv("heterozygosity_by_sample.tsv", sep="\t", header=T)

cthet$ct <- as.factor(cthet$ct)
cthet$md <- as.factor(cthet$md)
cthet$Heterozygosity <- cthet$Heterozygosity*100
cthet$Paralog_fraction <- cthet$Paralog_fraction*100
print(summary(cthet))

stats <- read.csv("basic_stats.tsv", header=T, sep="\t")
ct_factor <- as.factor(stats$ct)
stats$md <- as.factor(stats$md)
print(summary(stats))


# Read in and pre-process VCF files for PCoA

vcf_input_path_89 <- "../1_Lauf1/md10ct89ms13.vcf"
vcf_input_path_91 <- "../1_Lauf1/md10ct91ms13.vcf"
vcf_input_path_93 <- "../1_Lauf1/md10ct93ms13.vcf"

groups <- read.table("../1_Lauf1/continents.txt", header=T, sep="\t")

dat_89 <- read.vcfR(vcf_input_path_89)
dat_91 <- read.vcfR(vcf_input_path_91)
dat_93 <- read.vcfR(vcf_input_path_93)

# convert vcf data to genlight object; automatically omitts non-bialleic loci
dat.pre_gl_89 <- vcfR2genlight(dat_89)
ploidy(dat.pre_gl_89) <- 2
dat.gl_89 <- utils.reset.flags(dat.pre_gl_89, set=FALSE, verbose=0)
```

```
dat.pre_gl_91 <- vcfR2genlight(dat_91)

ploidy(dat.pre_gl_91) <- 2

dat.gl_91 <- utils.reset.flags(dat.pre_gl_91, set=FALSE, verbose=0)


dat.pre_gl_93 <- vcfR2genlight(dat_93)

ploidy(dat.pre_gl_93) <- 2

dat.gl_93 <- utils.reset.flags(dat.pre_gl_93, set=FALSE, verbose=0)



```

Fraction of loci inferred as paralogs and discarded by ipyrad across different clustering thresholds and minimum read depths

```{r Paralogs}
ggplot(cthet, aes(x=ct, y=Paralog_fraction)) +

  geom_boxplot(aes(fill=md),

        width=0.5,

        # custom boxes

        color="black",

        alpha=0.2,


        # custom outliers

        outlier.colour="darkgrey",

        outlier.fill="darkgrey",

        outlier.size=3


  ) +

  labs(x="Clustering threshold (% similarity)", y = "% flagged paralogs")+

  theme_classic() +

  theme(axis.text.x = element_text(angle=0, size=12)) +

  theme(axis.text.y = element_text(angle=90, size=12))
```

Heterozygosity across samples recovered across different clustering thresholds and minimum read depths

```{r Heterozygosity}
ggplot(cthet, aes(x=ct, y=Heterozygosity)) +
  geom_boxplot(aes(fill=md),
        width=0.5,
        # custom boxes
        color="black",
        alpha=0.2,

        # custom outliers
        outlier.colour="darkgrey",
        outlier.fill="darkgrey",
        outlier.size=3

  ) +
  labs(x="Clustering threshold (% similarity)", y = "% heterozygous sites")+
  theme_classic() +
  theme(axis.text.x = element_text(angle=0, size=12)) +
  theme(axis.text.y = element_text(angle=90, size=12))
```

Total SNPs recovered across different clustering thresholds and minimum read depths

```{r SNPs}
ggplot(stats, aes(x=ct, y=SNPs, color=md)) + geom_point() +
   labs(x="Clustering threshold (% similarity)", y = "Total SNPs") +
   theme_classic() +
   theme(axis.text.x = element_text(angle=0, size=12)) +
   theme(axis.text.y = element_text(angle=90, size=12)) +
   scale_x_continuous(breaks=seq(81,100,2))
```

Cumulative variance of all biallelic SNPs recovered by ipyrad explained by the first five principal components across different clustering thresholds and minimum read depths

```{r Var_expl}
ggplot(stats, aes(x=ct, y=VarExp5PCs, color=md)) + geom_point() +
    labs(x="Clustering threshold (% similarity)", y = "Cumulative variance in first 5 PCs") +
    theme_classic() +
    theme(axis.text.x = element_text(angle=0, size=12)) +
    theme(axis.text.y = element_text(angle=90, size=12)) +
    scale_x_continuous(breaks=seq(81,100,2))
```

Pearson's correlation coefficient between pairwise genetic dissimilarity and data missingness at different clustering thresholds and minimum read depths

```{r PCC}
ggplot(stats, aes(x=ct, y=PCCs, color=md)) + geom_point() +
    labs(x="Clustering threshold (% similarity)", y = "PCC between genetic distance and missingness") +
    theme_classic() +
    theme(axis.text.x = element_text(angle=0, size=12)) +
    theme(axis.text.y = element_text(angle=90, size=12)) +
    scale_x_continuous(breaks=seq(81,100,2)) +
    scale_y_continuous(breaks=seq(0.55,0.90,0.05))
```

Total number of loci that contain at least one SNP recovered across different clustering thresholds and minimum read depths

```{r Loci}
ggplot(stats, aes(x=ct, y=total_loci, color=md)) + geom_point() +
    labs(x="Clustering threshold (% similarity)", y = "Loci") +
    theme_classic() +
```

    theme(axis.text.x = element_text(angle=0, size=12)) +
    theme(axis.text.y = element_text(angle=90, size=12)) +
    scale_x_continuous(breaks=seq(81,100,2))
```

# % of differences between replicated samples across different clustering thresholds and minimum read depths

```{r Replicates}
ggplot(stats, aes(x=ct, y=rel_N_diff_replicates, color=md)) + geom_point() +
  labs(x="Clustering threshold (% similarity)", y = "% of differences
    between replicated samples;
    N = difference") +
  theme_classic() +
  theme(axis.text.x = element_text(angle=0, size=12)) +
  theme(axis.text.y = element_text(angle=90, size=12)) +
  scale_x_continuous(breaks=seq(81,100,2))
```

PCoA for clustering threshold 89% and minimum read depth 10

```{r PCoA_ct89, message = FALSE, warning = FALSE}

pc_89 <- gl.pcoa(dat.gl_89, nfactors=5, verbose=0)
# barplot(pc$eig/sum(pc$eig)*100, )
# explvar <- pc$eig/sum(pc$eig)*100
# cat("Explained variance:", explvar)
pcout_89 <- as.data.frame(pc_89$scores)
# plot(pcout$PC1, pcout$PC2)

ggplot(pcout_89, aes(x=PC1, y=PC2)) +
  geom_point(aes(shape=groups$Continent), size=2) +
  labs(x="Principal Component Axis 1", y = "Principal Component Axis 2") +

  theme_classic() +
  theme(axis.text.x = element_text(angle=0, size=12)) +
  theme(axis.text.y = element_text(angle=90, size=12))

```

PCoA for clustering threshold 91% and minimum read depth 10

```{r PCoA_ct91, message = FALSE, warning = FALSE}

pc_91 <- gl.pcoa(dat.gl_91, nfactors=5, verbose=0)
# barplot(pc$eig/sum(pc$eig)*100, )
# explvar <- pc$eig/sum(pc$eig)*100
# cat("Explained variance:", explvar)
pcout_91 <- as.data.frame(pc_91$scores)
# plot(pcout$PC1, pcout$PC2)

ggplot(pcout_91, aes(x=PC1, y=PC2)) +
  geom_point(aes(shape=groups$Continent), size=2) +
  labs(x="Principal Component Axis 1", y = "Principal Component Axis 2") +
  theme_classic() +
  theme(axis.text.x = element_text(angle=0, size=12)) +
  theme(axis.text.y = element_text(angle=90, size=12))

```

PCoA for clustering threshold 93% and minimum read depth 10

```{r PCoA_ct93, message = FALSE, warning = FALSE}

pc_93 <- gl.pcoa(dat.gl_93, nfactors=5, verbose=0)
# barplot(pc$eig/sum(pc$eig)*100, )

````
explvar <- pc$eig/sum(pc$eig)*100

cat("Explained variance:", explvar)

pcout_93 <- as.data.frame(pc_93$scores)

plot(pcout$PC1, pcout$PC2)

ggplot(pcout_93, aes(x=PC1, y=PC2)) +

  geom_point(aes(shape=groups$Continent), size=2) +

  labs(x="Principal Component Axis 1", y = "Principal Component Axis 2") +

  theme_classic() +

  theme(axis.text.x = element_text(angle=0, size=12)) +

  theme(axis.text.y = element_text(angle=90, size=12))


```
````
````
